# 744 Characteristics of Verified and Designated Burn Centers

**DOI:** 10.1093/jbcr/irad045.219

**Published:** 2023-05-15

**Authors:** Jeffrey Carter, Randy Kearns, Elle Lovick, Erica Murata, Bart Phillips

**Affiliations:** University Medical Center New Orleans & LSUHSC, River Ridge, Louisiana; University of New Orleans, New Orleans, Louisiana; University Medical Center- New Orleans Burn Unit, New Orleans, Louisiana; BData, Chicago, Illinois; BData Inc, Minneapolis, Minnesota; University Medical Center New Orleans & LSUHSC, River Ridge, Louisiana; University of New Orleans, New Orleans, Louisiana; University Medical Center- New Orleans Burn Unit, New Orleans, Louisiana; BData, Chicago, Illinois; BData Inc, Minneapolis, Minnesota; University Medical Center New Orleans & LSUHSC, River Ridge, Louisiana; University of New Orleans, New Orleans, Louisiana; University Medical Center- New Orleans Burn Unit, New Orleans, Louisiana; BData, Chicago, Illinois; BData Inc, Minneapolis, Minnesota; University Medical Center New Orleans & LSUHSC, River Ridge, Louisiana; University of New Orleans, New Orleans, Louisiana; University Medical Center- New Orleans Burn Unit, New Orleans, Louisiana; BData, Chicago, Illinois; BData Inc, Minneapolis, Minnesota; University Medical Center New Orleans & LSUHSC, River Ridge, Louisiana; University of New Orleans, New Orleans, Louisiana; University Medical Center- New Orleans Burn Unit, New Orleans, Louisiana; BData, Chicago, Illinois; BData Inc, Minneapolis, Minnesota

## Abstract

**Introduction:**

Burn centers serve an essential role in providing specialized care for the injured. Professional societies (verify) and state agencies (designate) these facilities through a process that evaluates the resources, processes, and capabilities of each burn center. Over 90% of the level 1 or 2 trauma centers in the US and only around 50% of burn centers are verified. The goal of our study was to evaluate the variability of verified and designated burn centers and trauma centers in the US.

**Methods:**

The Databases for Optimal Resources for Injury Care (DORIC) is a centralized database developed by the American Hospital Association, American College of Surgeons Committee on Trauma, American Burn Association, all fifty US states’ Departments of Health, and claims databases through a collaboration with BData, LSUHSC, Spectral MD, and UNO. Categorical data was assessed using Fisher’s exact while continuous variables were assessed with paired t-test and confidence intervals. The analysis included hospital and burn ICU bed count, hospital revenue, ED visits, discharges, and urban vs rural geography.

**Results:**

The database includes 134 burn centers of which 75 were verified (56%), 91% were in an urban setting and 87% were co-located with a trauma center. Hospitals had a median of 492 staffed beds, 79,449 ER visits, and 18,437 discharges. Burn centers had a median of 11 burn ICU beds and 3,285 hospital days/year. Urban vs rural geography was not different between verified and designated burn centers (*p*=0.54). All categories of revenue were associated with burn center verification.

**Conclusions:**

Our study demonstrates no significant difference with the hospital size, location (urban vs rural), ER volume, hospital beds, or burn ICU beds. Significant differences were identified with the rate of discharge and revenues which could be attributed to services, quality, or reimbursement. Given that only half of burn care occurs at verified burn centers, additional research is needed to better understand these significant differences.

**Applicability of Research to Practice:**

A keen understanding of burn center characteristics is essential to serve the burn professionals that work at burn centers and the communities they serve.

